# Design, Modeling, and Control of a New Multi-Motion Mobile Robot Based on Spoked Mecanum Wheels

**DOI:** 10.3390/biomimetics8020183

**Published:** 2023-04-28

**Authors:** Jie Leng, Haiming Mou, Jun Tang, Qingdu Li, Jianwei Zhang

**Affiliations:** 1 School of Optoelectronic Information and Computer Engineering, University of Shanghai for Science and Technology, Shanghai 200093, China; 191550059@st.usst.edu.cn (J.L.);; 2 Department of Informatics, University of Hamburg, 20146 Hamburg, Germany

**Keywords:** multi-movement mode, mobile robots, spoked mecanum wheel

## Abstract

This paper presents an exciting and meaningful design to make mobile robots capable of adapting to various terrains. We designed a relatively simple and novel composite motion mechanism called the flexible spoked mecanum (FSM) wheel and created a mobile robot, LZ-1, with multiple motion modes based on the FSM wheel. Based on the motion analysis of the FSM wheel, we designed an omnidirectional motion mode for this robot, allowing it to move flexibly in all directions and successfully traverse rugged terrains. In addition, we designed a crawl motion mode for this robot, which can climb stairs effectively. We used a multilayer control method to move the robot according to the designed motion modes. Multiple experiments showed that these two motion modes for the robot are effective on various terrains.

## 1. Introduction

Mobile robots play an increasingly important role in human production activities and day-to-day life. Various mobile robots such as legged, tracked, wheeled, and hybrid robots have been designed, among which wheeled mobile robots have attracted the most attention due to their potential applications in warehousing, logistics, environmental monitoring, agriculture, etc. However, since wheeled mobile robots have a simple structure and are easy to control, they are only suitable for flat ground and cannot be applied to complex surfaces such as stairs and rough roads. In order to adapt to complex terrains, many types of mobile robots have been developed, such as quadruped and biped robots, among which the Cheetah robot [1] of MIT and the Cassie robot [2] of the University of Michigan are the most typical. This kind of legged robot has good locomotion on complex terrains, such as grass, rugged fields, and stairs, but its moving efficiency on flat ground is much lower than that of wheeled robots. The high cost, complicated mechanisms, and challenging control design hinder these robots’ popularization and use. To make the mobile robots have excellent locomotion and mobile efficiency simultaneously, researchers have also proposed a variety of robots with hybrid motion modes to make these mobile robots consider the efficiency of plane movement and the trafficability of complex terrain.

Hybrid mobile robots can be generally divided into two categories: the first one is wheel-legged hybrid robots that connect wheels and legs, and the other is mobile robots that have varying wheels or leg structures that can change shape. The first category is a direct combination of wheels and legs. Jianwei Zhao et al. [3] developed a four-legged hybrid robot with wheels at the end of each leg. It can use the single-joint motion of its two front legs to overcome obstacles and use the wheels to realize fast motion on flat ground. Ernesto C. et al. [4] proposed a hybrid wheel-legged hexapod robot, Cassino Hexapod III, using mecanum wheels. They studied the legged locomotion, wheeled locomotion, and wheel–leg locomotion of the robot in detail and achieved the leg motion planning, omnidirectional wheeled motion, and obstacle-crossing motion of the wheel–leg combination. Zhihua Chen et al. [5] studied a hybrid obstacle-avoidance gait for a six-wheel-legged robot, BIT-6NAZA, in detail. When the robot moves on flat ground, it mainly relies on rolling the wheels at the ends of the legs. When encountering obstacles, the legs avoid or cross the obstacles according to the designed gait. Zhihua Chen et al. [6] further proposed a hierarchical control framework to enable BIT-6NAZA to plan a flexible gait on unstructured roads according to terrain feedback information, which adapts to terrain changes. Additionally, Shoukun Wang et al. [7] proposed a hierarchical framework that integrates wheel speed, leg motion planning, and a whole-body control framework. These frameworks allow the BIT-6NAZA to maintain its horizontal stability while traversing obstacles in different terrains. The second category is a redesign of the locomotion mechanism by integrating the features of wheels and legs. The bionic robot RHex [8] is a typical example, and its locomotion mechanism is essentially a rimless wheel with a single spoke. Whegs [9] are upgraded versions of rimless wheels with four spokes. Rimless or spoked wheels rotate like standard wheels and use discrete spokes to achieve leg-like movement. Mingyuan Yang et al. [10] proposed a hexapod robot with whegs driven by a motor. It has a simple structure and excellent locomotion, but cannot produce steering movement. Its spoked wheels have a remarkable obstacle-crossing ability, but the discrete spokes also make the robot’s movement irregular. Yuan Tao et al. [11] proposed a transformable wheel mechanism that can be transformed between a three-spoked rimless wheel and a standard wheel structure to give the mobile robot good obstacle-crossing ability and mobility. Ruixiang Cao et al. [12] designed a transformable omniwheel mechanism that can be transformed between a three-spoked rimless wheel and an omniwheel structure with three spokes, so the mobile robot can cross obstacles and move in all directions. Cunxi Dai et al. [13] adopted a similar idea to design a SWheg that can change between an S-shaped, two-spoked wheg and a wheel configuration.

Although the first kind of wheel-legged hybrid robot has the movement ability of both legs and wheels, the overall structure is still very complicated, and the manufacturing cost is high. It is also tough to model and control its wheel–leg locomotion. Another kind of hybrid robot with spoked wheels is simple in structure and control, but it needs more smoothness and flexibility in wheeled movement. While the transformed whegs improve the performance of the spoked wheels, these designs are still complicated, and the transformation process introduces some unexpected actions.

**Figure 1:**
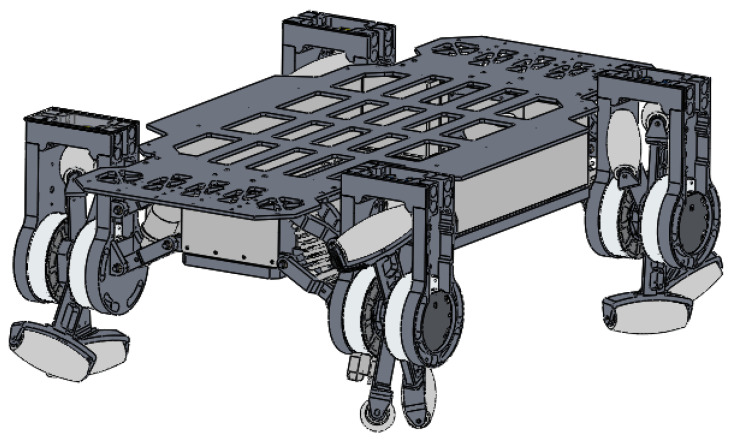
The mobile robot LZ-1.

Motivated by the above observations, this paper proposes a mobile robot, LZ-1, which is suitable for various terrains and has a simple structure. The robot is equipped with a new motion mechanism: a flexible spoked mecanum (FSM) wheel. The design of the FSM wheel draws from the structure of the oblique rollers of standard mecanum wheels, and similar rollers are installed at the ends of the spokes, which makes the FSM wheel have oblique movement. At the same time, the four spokes of the FSM wheel can rotate around the wheel axis independently, which is similar to the design in [14]. Based on the unique structure design of the FSM wheel, we designed a novel movement mode that made the ends of the four spokes alternately contact the ground so that the FSM wheel had not only the flexibility of mecanum wheels, but also the locomotion of whegs [9]. Figure 1 shows the overall structure of the LZ-1 mobile robot. LZ-1 has a similar structure design to standard mecanum-wheeled robots. It has a rectangular rigid main body with four FSM wheels mounted on its four corners. These four FSM wheels are divided into two types, which differ in angle between the roller and wheel axes. Our design used $60^{\circ }$ and $-60^{\circ }$ for these two types (the angle values in the later part of this article are in degrees, not radians). The same two FSM wheels were installed diagonally, which conforms to the design rule of mecanum-wheeled robots. This design allows the LZ-1 to generate velocity in any direction on the horizontal plane. We designed two gaits for the LZ-1 robot to enable, respectively, omnidirectional movement and climbing stairs and evaluated the actual effects of these two gaits through the corresponding experiments. The main contributions of this work are summarized as follows:

1. A simple-structured flexible spoked mecanum wheel with excellent obstacle-crossing and omnidirectional movement capabilities is proposed. In addition, a mobile robot, LZ-1, based on this motion mechanism was designed and manufactured for experimentation.
2. An omnidirectional motion mode that can achieve omnidirectional movement and surpasses the obstacle-crossing performance of traditional mecanum wheel mobile robots and a crawl motion mode that can climb continuous stairs were developed for the LZ-1 robot.
3. Numerous experiments demonstrated that the omnidirectional motion mode and crawl motion mode developed for the LZ-1 robot are practical and can be used in future mobile operations on unstructured terrains.

## 2. Design, Modeling, and Control

### 2.1. Robot Design

**Figure 2:**
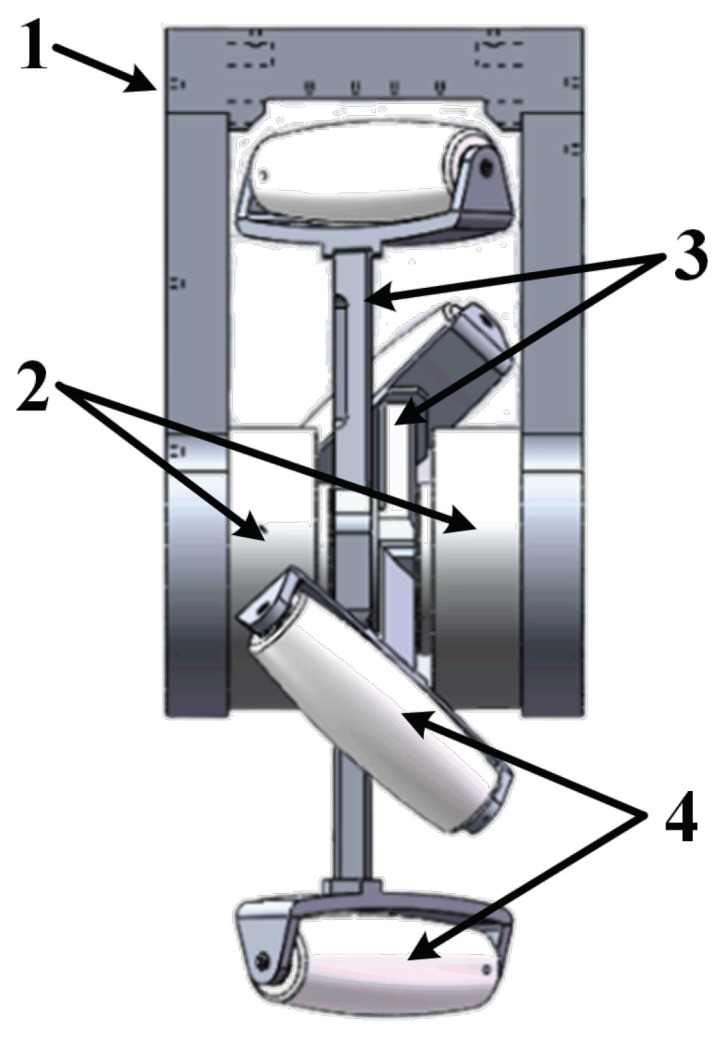
Side view of the flexible spoked mecanum wheel. In the figure, numeral 1 denotes the U-shaped metal piece, numeral 2 denotes the servo motor, numeral 3 denotes the spoke component, and numeral 4 denotes the roller.

As a critical component of the LZ-1 robot, we introduce the FSM wheel in detail. Figure 2 shows the side views of the FSM wheel. As shown in Figure 2, the FSM wheel consists of four parts:

1. A U-shaped metal piece for fixing two spoke components and connecting the FSM wheel to the robot’s main body;
2. Two servo motors that drive the two spoke components separately. These servo motors are fixed on that U-shaped piece, and their rotation axes are collinear. Each servo motor has a built-in controller that allows us to easily set its speed or rotation position through a communication bus;
3. Two spoke components with their centers fixed on the servo motor rotation axes;
4. Four rollers that can rotate freely under external force at each end of each spoke component.

We used a high-strength aluminum alloy to make the spoke components because they directly bear the robot’s weight and forces from the ground during movement. The rollers are rubber, which can reduce the impact force to some extent and improve the service life. We also used ball bearings at the roller shafts to reduce friction when the rollers rotate.

**Figure 3:**
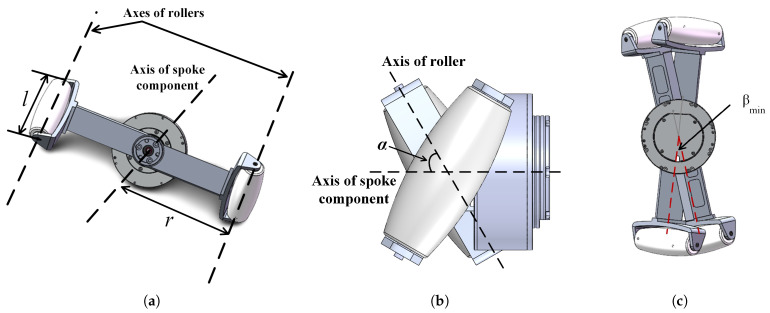
Details of the spoke component. (**a**) The spoke component marked with axes. (**b**) Top view of the spoke component. (**c**) Limit of included angle between spokes.

The two spoke components in the FSM wheel are the most essential. The way they move together can form different movement modes, which we explain in Section 2.2. A single spoke component provides two spokes $180^{\circ }$ apart, as shown in Figure 3a. The configuration of the rollers at both ends of the spoke component is similar to that of the rollers around the hub in a traditional mecanum wheel; that is, there is an angle α between the axis of the spoke component and that of the roller, as shown in Figure 3b. The length of a spoke is half that of the spoke component and is denoted by *r*. The axial length of a roller is denoted by *l*. The diameter of a cross-section through the center of a roller is denoted by *d*. When both motors in an FSM wheel rotate synchronously, all rollers move with a circular trajectory centered at the center of the FSM wheel and radius $r+\frac{1}{2}d$. It is worth noting that the two spoke components are mutually constrained during motion and cannot rotate freely past each other; a minimum angle exists between them. Figure 3c shows the ultimate values for the angles between the two spoke components due to physical constraints; angle $\beta_{min}$, marked by two red dashed lines, represents the minimum angle between the two spoke components. Due to symmetry in the FSM wheel structure, the angle at the other ends between the two spoke components has the maximum value $\beta_{max}$. $\beta_{min}$ and $\beta_{max}$ satisfy the relationship: $\beta_{min}+\beta_{max}=180$. Table 1 lists the fundamental parameter values for our research on the FSM wheels.

**Figure 4:**
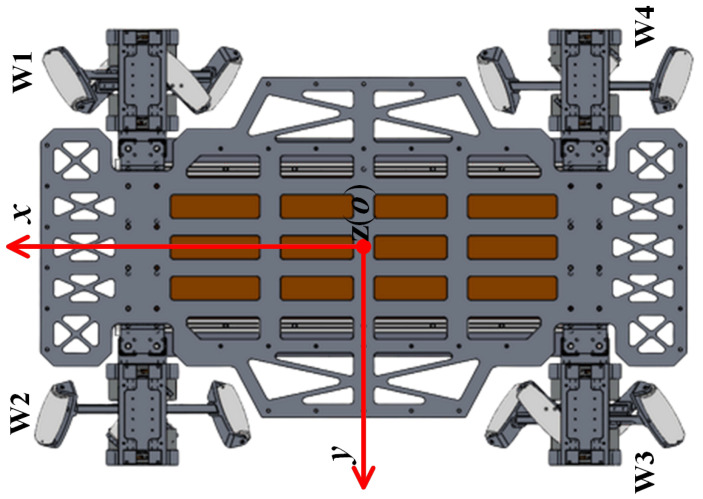
The top view of the LZ-1 robot. W1, W2, W3, and W4, respectively, represent the numbers of the four FSM wheels.

The overall configuration of the LZ-1 mobile robot is similar to that of a standard mecanum-wheeled mobile robot. The main difference is that the LZ-1 robot uses FSM wheels instead of mecanum wheels. The LZ-1 robot consists of four FSM wheels and one rigid body, as shown in Figure 1. There are two ways to arrange mecanum wheels for standard mecanum-wheeled mobile robots [15,16]. We adopted a method similar to the Class A layout in [16] to deploy four FSM wheels at the four corners of the LZ-1’s body. The FSM wheels with positive α values were deployed on the main diagonal of the LZ-1’s body, while those with negative α values were deployed on the secondary diagonal. We connected the FSM wheel to the robot body through a shock absorber with a spring, which can somewhat reduce the impact from the ground on the LZ-1’s body. The main body of the LZ-1 is a cuboid composed of rigid support plates, batteries, main controllers, power management modules, and rigid cover plates. Figure 4 shows the top view of the entire robot, which is rotationally symmetrical around its geometric center. Table 2 lists the critical parameters for the LZ-1. In this table, the height of the LZ-1 refers to the maximum distance from the top of the LZ-1 to the flat ground in contact with the FSM wheels. The distance between the front and rear FSM wheels and the distance between the left and right FSM wheels refer to the distance between the geometric centers of the two FSM wheels.

The hardware design of the LZ-1’s controller adopted a hierarchical approach, divided into a main control layer and a motor control layer. The main control layer mainly provides hardware support for functions such as gait planning, perception information aggregation, and remote control signal reception for the robot. The motor control layer consists of local controllers for all the servo motors in the FSM wheels and provides hardware support for motor speed or position control. The two layers communicate through an RS-485 serial communication bus. Section 2.3 describes the controllers in detail.

### 2.2. Locomotion Modeling

The FSM wheel has two spoke components driven by motors, which can contact the ground in various ways and produce a variety of motion modes. The LZ-1 is equipped with four FSM wheels, and different FSM wheel motion modes result in more diverse motion effects for the LZ-1. In this article, we mainly discuss two fundamental motion modes of the LZ-1, namely:

1. Omnidirectional motion mode: In this mode, the LZ-1 can achieve omnidirectional movement on flat ground, similar to a mecanum-wheeled mobile robot, while being able to cross some concave or convex obstacles.
2. Crawl motion mode: In this mode, the LZ-1 can climb standard walking stairs.

Next, we analyze how these two motion modes are realized and design the motion control method of the LZ-1.

#### 2.2.1. Omnidirectional Motion Mode

The FSM wheel has two spoke components that can move independently and limit each other’s motion range. For the LZ-1 to achieve omnidirectional movement like a mecanum-wheeled mobile robot, the simplest way is to use an FSM wheel to simulate the movement of a traditional mecanum wheel. G. Wampfler et al. analyzed the movement mechanism of mecanum wheels in detail [17]. When a traditional mecanum wheel moves on a plane, only one roller is generally in contact with the plane. Only when the roller in contact with the ground switches with the adjacent roller due to the rotation of the hub are the two rollers in contact with the ground at the same time, and this time frame is very short in the normal movement of mecanum-wheeled robots. In other words, when the mecanum wheel rotates on the plane due to the drive of the motor, the rollers fixed around the hub come into contact with the plane in turn. By planning the movement trajectories of the two spoke components of the FSM wheel reasonably, we can achieve the movement mentioned above of the mecanum wheel and then use the inverse kinematics equation of the mecanum-wheeled mobile robot to solve the corresponding rotation speeds $w_{n}$ (*n* = 1, 2, 3, or 4) (unit deg/s) of the four FSM wheels for the given velocity ${\vec{v}}_{r}$ of the LZ-1 so that we can realize its omnidirectional movement. The number distribution of these four FSM wheels on the LZ-1 is shown in Figure 4.

**Figure 5:**
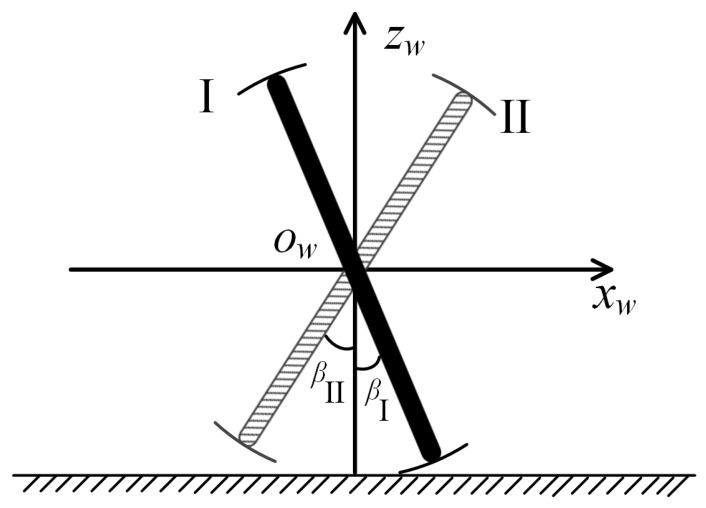
A simple geometric model of an FSM wheel.

In order to facilitate the description of the trajectory planning of the two spoke components in the FSM wheel, we used a simplified two-dimensional geometric model of the FSM wheel, as shown in Figure 5. We used a rectangular rod to represent the spoke component of the FSM wheel and an arc with a radius of $r+\frac{d}{2}$ and an angle of $\beta_{min}$ to represent the roller. In Figure 5, the black rectangular rod with arc lines at both ends is called Spoke Component I, and the gray rectangular rod with arc lines at both ends is named Spoke Component II. The $z_{w}$ axis of the coordinate system $x_{w}o_{w}z_{w}$ is perpendicular to the ground plane. The $x_{w}$ axis is parallel to the ground plane and points to the right side of the FSM wheel’s geometric model, and the origin $o_{w}$ is the center of the two spoke components. $\beta_{I}$ is the angle between Spoke Component I and the $z_{w}$ axis, and $\beta_{II}$ is the angle between Spoke Component II and the $z_{w}$ axis. Due to the structural symmetry of the FSM wheel, $\beta_{I}$ and $\beta_{II}$ have a range of 180, which can describe all the positions of the corresponding spoke component. In order to facilitate the subsequent description of the trajectory planning of the spoke component, we took $\beta_{I},\beta_{II}\in \left[-\frac{\beta_{min}}{2},180-\frac{\beta_{min}}{2}\right]$. When $\beta_{I}=-\frac{\beta_{min}}{2}$, Spoke Component I has the same configuration as when $\beta_{I}=180-\frac{\beta_{min}}{2}$; that is, $\beta_{I}$ is equivalent at its value boundary. Similarly, $\beta_{II}$ is also equivalent at its value boundary.

**Figure 6:**
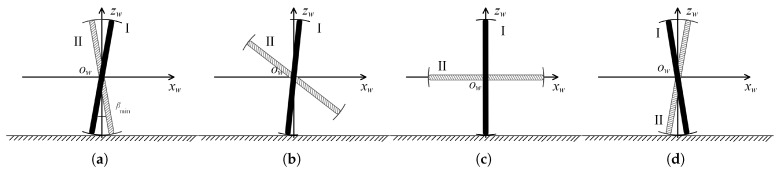
The FSM wheel realizes the motion process of a mecanum wheel. (**a**) Start of a cycle. (**b**) A single roller touches the ground during the cycle. (**c**) The position of the spoke components in the middle of the cycle. (**d**) Start of next cycle.

We designed a cyclic motion process, as shown in Figure 6, to enable the FSM wheel to simulate the motion effect of the traditional mecanum wheel. Figure 6a shows the start of a cycle when both ends of the two spoke components are in contact with the ground and are rotating at a speed of $w_{n}$. At this time, the angle between the two spoke components is $\beta_{min}$, that is $\beta_{I}=-\frac{\beta_{min}}{2}$, $\beta_{II}=\frac{\beta_{min}}{2}$, which is the same as when two rollers of the mecanum wheel are in contact with the ground plane at the same time. When the two spoke components rotate in the given direction of the FSM wheel, one spoke component (here, Spoke Component I is used as an example) stays in contact with the ground and rotates at a speed of $w_{n}$, which we call the supporting spoke. In contrast, the other spoke component (Spoke Component II in the figure) leaves the ground and rotates at a suitable speed, which we call the swinging spoke, as shown in Figure 6b. For convenience, we denote the angle between the supporting spoke and the $z_{w}$ axis as $\beta_{s}$ and the angle between the swinging spoke and the $z_{w}$ axis as $\beta_{w}$. In the example, $\beta_{s}=\beta_{I}$ and $\beta_{w}=\beta_{II}$. The stage shown in Figure 6c is similar to that shown in Figure 6b, where the supporting spoke is perpendicular to the ground, and its rotation speed is still $w_{n}$. The swinging spoke is parallel to the ground and rotates at a suitable speed. Figure 6d shows the end of this movement cycle, where the supporting spoke is still in contact with the ground, $\beta_{s}=\frac{\beta_{min}}{2}$, and its rotation speed is still $w_{n}$; the swinging spoke contacts with the ground again, $\beta_{w}=-\frac{\beta_{min}}{2}$, and its rotation speed is $w_{n}$. The motion of the two spoke components of the FSM wheel repeats the above process, but in the next cycle, the supporting and swinging spoke are switched. In the example, Spoke Component I becomes the swinging spoke and Spoke Component II becomes the supporting spoke. Throughout the process, the rotation speed of the supporting spoke is always $w_{n}$, and $\beta_{s}\in \left[-\frac{\beta_{min}}{2},\frac{\beta_{min}}{2}\right]$ ensures that the center of the FSM wheel always stays at a height of $r+\frac{1}{2}d$ from the ground. At the same time, the rotation speed of the swinging spoke changes according to the suitable plan, but this does not affect the motion effect of the FSM wheel on the plane. The whole motion process is the same as when a single roller of the mecanum wheel contacts with the ground and when two rollers contact with the ground at the same time, and the motion effects of the mecanum wheel and the FSM wheel are both caused by the rollers in contact with the ground, which ensures that the FSM wheel can simulate the same motion effect on the plane as the traditional mecanum wheel.

In order to make the swinging spoke rotate to follow the above motion process while ensuring the continuity and smoothness of the motor’s acceleration to improve its service life, we used a fifth-order polynomial trajectory planning method to generate a suitable motion trajectory for the swinging spoke. Before that, we describe the motion equation of the supporting spoke in one cycle. According to the above motion process description, $\beta_{s}$ is a linear function of time *t* in one cycle, and its slope is $w_{n}$. For the convenience of calculation, we took a moment when $\beta_{s}=0$ as t=0, that is $\beta_{s}\left(0\right)=0$, so we have

(1) $$\begin{array}{c} \beta_{s}=\beta_{s}\left(t\right)=w_{n}\left(t+\frac{\beta_{min}}{2w_{n}}\right)-\frac{\beta_{min}}{2},-\frac{\beta_{min}}{2}\leq \beta_{s}\left(t\right)<\frac{\beta_{min}}{2} \end{array}$$

where $w_{n}\neq 0$. When $w_{n}=0$, the FSM wheel does not move. From Equation 1, we can obtain $t=\beta_{s}/w_{n}$.

Assuming that, within the same period, the relationship between $\beta_{w}$ and time *t* satisfies a fifth-order polynomial,

(2) $$\begin{array}{c} \beta_{w}=\beta_{w}\left(t\right)=C_{0}+C_{1}t+C_{2}t^{2}+C_{3}t^{3}+C_{4}t^{4}+C_{5}t^{5},\frac{\beta_{min}}{2}\leq \beta_{w}<180-\frac{\beta_{min}}{2} \end{array}$$

The first derivative of Equation (2) concerning *t*, that is the rotational speed of the swinging spoke, is

(3) $$\begin{array}{c} \dot{\beta_{w}\left(t\right)}=C_{1}+2C_{2}t+3C_{3}t^{2}+4C_{4}t^{3}+5C_{5}t^{4} \end{array}$$

The second derivative of Equation (2) concerning *t*, that is the angular acceleration of the swinging spoke, is

(4) $$\begin{array}{c} \ddot{\beta_{w}\left(t\right)}=2C_{2}+6C_{3}t+12C_{4}t^{2}+20C_{5}t^{3} \end{array}$$

According to the above-designed FSM wheel motion planning, it can be known that

(5) $$\frac{\beta_{min}}{2}=C_{0}+C_{1}\left(-\frac{\beta_{min}}{2w_{n}}\right)+C_{2}{\left(-\frac{\beta_{min}}{2w_{n}}\right)}^{2}+C_{3}{\left(-\frac{\beta_{min}}{2w_{n}}\right)}^{3}+C_{4}{\left(-\frac{\beta_{min}}{2w_{n}}\right)}^{4}+C_{5}{\left(-\frac{\beta_{min}}{2w_{n}}\right)}^{5}$$

(6) $$180-\frac{\beta_{min}}{2}=C_{0}+C_{1}\frac{\beta_{min}}{2w_{n}}+C_{2}{\left(\frac{\beta_{min}}{2w_{n}}\right)}^{2}+C_{3}{\left(\frac{\beta_{min}}{2w_{n}}\right)}^{3}+C_{4}{\left(\frac{\beta_{min}}{2w_{n}}\right)}^{4}+C_{5}{\left(\frac{\beta_{min}}{2w_{n}}\right)}^{5}$$

(7) $$w_{n}=C_{1}+2C_{2}\left(-\frac{\beta_{min}}{2w_{n}}\right)+3C_{3}{\left(-\frac{\beta_{min}}{2w_{n}}\right)}^{2}+4C_{4}{\left(-\frac{\beta_{min}}{2w_{n}}\right)}^{3}+5C_{5}{\left(-\frac{\beta_{min}}{2w_{n}}\right)}^{4}$$

(8) $$w_{n}=C_{1}+2C_{2}\frac{\beta_{min}}{2w_{n}}+3C_{3}{\left(\frac{\beta_{min}}{2w_{n}}\right)}^{2}+4C_{4}{\left(\frac{\beta_{min}}{2w_{n}}\right)}^{3}+5C_{5}{\left(\frac{\beta_{min}}{2w_{n}}\right)}^{4}$$

(9) $$0=2C_{2}+6C_{3}\left(-\frac{\beta_{min}}{2w_{n}}\right)+12C_{4}{\left(-\frac{\beta_{min}}{2w_{n}}\right)}^{2}+20C_{5}{\left(-\frac{\beta_{min}}{2w_{n}}\right)}^{3}$$

(10) $$0=2C_{2}+6C_{3}\frac{\beta_{min}}{2w_{n}}+12C_{4}{\left(\frac{\beta_{min}}{2w_{n}}\right)}^{2}+20C_{5}{\left(\frac{\beta_{min}}{2w_{n}}\right)}^{3}.$$

By solving the above Equations (5)–(10), we find

(11) $$\left[\begin{array}{c} C_{0}\\ C_{1}\\ C_{2}\\ C_{3}\\ C_{4}\\ C_{5} \end{array}\right]=\left[\begin{array}{c} 90\\ \frac{-\left(11\beta_{min}-1350\right)w_{n}}{4\beta_{min}}\\ 0\\ \frac{10\left(\beta_{min}-90\right)w_{n}^{3}}{\beta_{min}^{3}}\\ 0\\ \frac{-12\left(\beta_{min}-90\right)w_{n}^{5}}{\beta_{min}^{5}} \end{array}\right]$$

Substituting this solution into Equation (2), we obtain

(12) $$\begin{array}{c} \beta_{w}=\beta_{w}\left(t\right)=90+\frac{-\left(11\beta_{min}-1350\right)w_{n}}{4\beta_{min}}t+\frac{10\left(\beta_{min}-90\right)w_{n}^{3}}{\beta_{min}^{3}}t^{3}+\frac{-12\left(\beta_{min}-90\right)w_{n}^{5}}{\beta_{min}^{5}}t^{5},\frac{\beta_{min}}{2}\leq \beta_{w}<180-\frac{\beta_{min}}{2}. \end{array}$$

So far, we have obtained the motion planning trajectory equations for the supporting and swinging spokes within one cycle. According to the planning of Equations (1) and (12), near the end of one motion cycle, the motions of the supporting spokes and the swinging spokes are close to their position boundaries. Due to the symmetry of the spoke components, the supporting spokes and swinging spokes naturally switch at the start of the following motion cycle.

**Figure 7:**
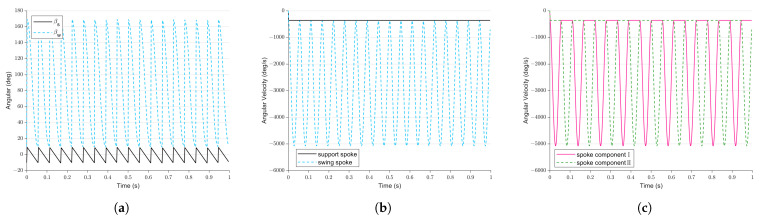
Motion of two spoke components of the FSM wheel in omnidirectional motion mode. (**a**) Change in angle of the support spoke and the swing spoke. (**b**) Change in angular velocity of the support spoke and the swing spoke. (**c**) Change in angle of the two spoke components.

According to the planning of Equations (1) and (12), Figure 7 demonstrates the motion changes produced by the two spoke components of the FSM wheel when $w_{n}=-360$ and $\beta_{min}=20$. Figure 7a,b show the angle and speed changes in the spokes from the perspective of the supporting and swinging spokes. We can see that the angles of the swinging spoke and supporting spoke change periodically and smoothly transition at the switching angles of $-10^{\circ }$ or $10^{\circ }$; the angular velocity of the supporting spoke is always consistent with $w_{n}$, and the angular velocity of the swinging spoke changes periodically and reaches its boundary value when $\beta_{w}=90$. Figure 7c shows that, due to the alternating changes in the supporting spoke and swinging spoke, the angular velocities of Spoke Component I and Spoke Component II also change periodically. The two are half a cycle apart.

Given the moving speed ${\vec{v}}_{r}$ of the LZ-1, the value of $w_{n}$ can be calculated by the inverse kinematics equation of the mecanum-wheeled mobile robot. According to the configuration of LZ-1, we used its body coordinate system xyz as shown in Figure 4. In the figure, the *x* axis is along the long axis of the robot body pointing towards the front of the robot, the *y* axis is along the short axis of the robot body pointing towards the left side of the robot, the *z* axis is perpendicular to the robot body pointing upwards, and the origin *o* is at the center of the robot and is coplanar with the axes of the four FSM wheels. The speed of the LZ-1 is ${\vec{v}}_{r}=\left(v_{rx},v_{ry},w_{rz}\right)$, where the component $v_{rx}$ is the speed of the LZ-1 moving along the *x* axis, the component $v_{ry}$ is the speed of the LZ-1 moving along the *y* axis, and the component $w_{rz}$ is the angular velocity of the LZ-1 rotating around the *z* axis (in rad/s). According to the kinematic relationship of the mecanum-wheeled mobile robot [18], in the omnidirectional motion mode of the LZ-1, $v_{rx}$, $v_{ry}$, $w_{rz}$, and $w_{n}\left(n=1,2,3,4\right)$ satisfy the following system of equations:

(13) $$\left[\begin{array}{c} w_{1}\\ w_{2}\\ w_{3}\\ w_{4} \end{array}\right]=\frac{180}{\pi (r+\frac{1}{2}d)}\;\cdot \;\left[\begin{array}{ccc} 1 & \frac{\sqrt{3}}{3} & 0.315\frac{\sqrt{3}}{3}+0.2445\\ 1 & -\frac{\sqrt{3}}{3} & -(0.315\frac{\sqrt{3}}{3}+0.2445)\\ 1 & \frac{\sqrt{3}}{3} & -(0.315\frac{\sqrt{3}}{3}+0.2445)\\ 1 & -\frac{\sqrt{3}}{3} & 0.315\frac{\sqrt{3}}{3}+0.2445 \end{array}\right]\left[\begin{array}{c} v_{rx}\\ v_{ry}\\ w_{rz} \end{array}\right]$$

Through Equation (13), we can easily calculate the rotation speed of the four FSM wheels under the planning of Equations (1) and (12) when the moving speed goal of the LZ-1 is given.

**Figure 8:**
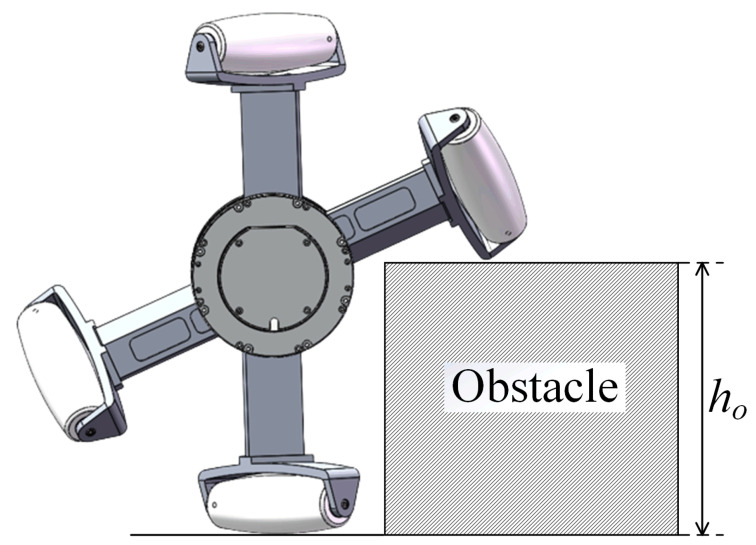
The schematic diagram of an FSM wheel encountering an obstacle.

Compared to the motion performance of the mecanum-wheeled mobile robot, the omnidirectional motion mode of the LZ-1 has a better obstacle-crossing ability. Because the number of spokes on the FSM wheel is sparser than that of the traditional mecanum wheel and the gap between the spokes of the FSM wheel is larger during motion, the FSM wheel can overcome taller obstacles, as shown in Figure 8. When the angle between the two spoke components of the FSM wheel is $\left|\beta_{I}-\beta_{II}\right|=\beta_{min}$, the FSM wheel reaches its theoretical limit of obstacle crossing, that is, the maximum value of $h_{o}$ is $\left(r+\frac{d}{2}\right)\left(1+cos\beta_{min}\right)\approx 0.345$ (in units of m). The maximum obstacle height that a traditional mecanum wheel of the same size can overcome is $r+\frac{d}{2}=0.178$, only half that of the FSM wheel. The above data show that the obstacle-crossing performance of the FSM wheel is far better than that of the traditional mecanum wheel.

#### 2.2.2. Crawl Motion Mode

Although the LZ-1’s omnidirectional motion mode has excellent obstacle-crossing ability, it cannot guarantee that the LZ-1 always stays in a state with maximum obstacle-crossing capacity, because the angle between the two spoke components of the FSM wheel changes periodically during this mode of motion. The LZ-1’s omnidirectional motion mode is not applicable when encountering continuous obstacles, such as stairs. In this case, keeping the FSM wheel in the state with maximum obstacle-crossing ability may work. Based on this idea, we designed a crawl motion mode for LZ-1. This mode is mainly used to traverse structured, non-flat surfaces such as stairs. When the LZ-1 is in the crawl motion mode, the FSM wheel’s motion model and the stair climbing principle are similar to those described in [19].

**Figure 9:**
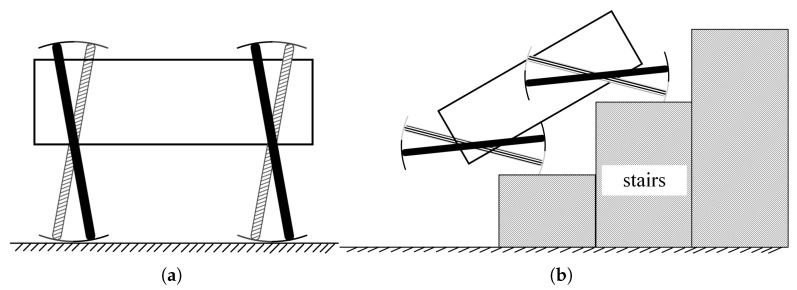
The crawl motion model of LZ-1. (**a**) The initial state of climbing motion mode of LZ-1. (**b**) Schematic diagram of LZ-1 climbing stairs in crawl motion mode.

As described in the previous section, when the angle between the two spoke components of the FSM wheel is $\beta_{min}$, the FSM wheel has the greatest obstacle-crossing ability. Therefore, in crawl motion mode, the four FSM wheels of the LZ-1 keep the angle between their spoke components as $\beta_{min}$. The four FSM wheels of the LZ-1 maintain the shape shown in Figure 3c and rotate synchronously. Figure 9a shows the initial state of the LZ-1 in crawl motion mode, where the four FSM wheels start to move synchronously with this shape. Figure 9b illustrates the LZ-1 climbing stairs in crawl motion mode. The synchronous movement of the four FSM wheels adapts to the structured stairs, and their eight motors are all used to support and lift the main body of the LZ-1, which is very effective in moving the robot over steep stair surfaces. According to this planning, at the initial time, the angle of Spoke Component I is $\beta_{I}=\frac{\beta_{min}}{2}$ and the angle of Spoke Component II is $\beta_{II}=-\frac{\beta_{min}}{2}$. In the motion process, the rotation speed of Spoke Components I and II satisfies the equation group:

(14) $$\begin{array}{c} \dot{\beta_{I}}=w_{n},\dot{\beta_{II}}=w_{n} \end{array}$$

where $w_{n}$ is consistent with the previous definition as the target speed of the FSM wheel and $w_{n}=\frac{180v_{rx}}{\pi (r+\frac{1}{2}d)}$, $w_{1}=w_{2}=w_{3}=w_{4}$. $\dot{\beta_{I}}$ and $\dot{\beta_{II}}$, respectively, represent the rotational speed of Spoke Components I and II (the unit is deg/s).

Because the four FSM wheels in the crawl motion mode of the LZ-1 rotate synchronously and at the same speed, the lateral movement trends of the rollers at the ends of all spokes cancel each other out, and the combined total movement results in the LZ-1 moving forward or backward along its *x* axis, that is ${\vec{v}}_{r}=\left(v_{rx},0,0\right)$. Therefore, when traversing stairs, this movement method can reduce the possibility of the LZ-1 slipping to the side.

### 2.3. Control Method

Using Equations (1) and (12)–(14), we can calculate the motion trajectories of the four FSM wheels in omnidirectional movement mode and crawl motion mode according to the given LZ-1 velocity ${\vec{v}}_{r}$. These motion trajectories show the time-related position or velocity sequences of the two spoke components in the FSM wheel. We can synchronize these sequences to the local controllers of the two spoke components’ servo motors to achieve the motion control of the LZ-1.

**Figure 10:**
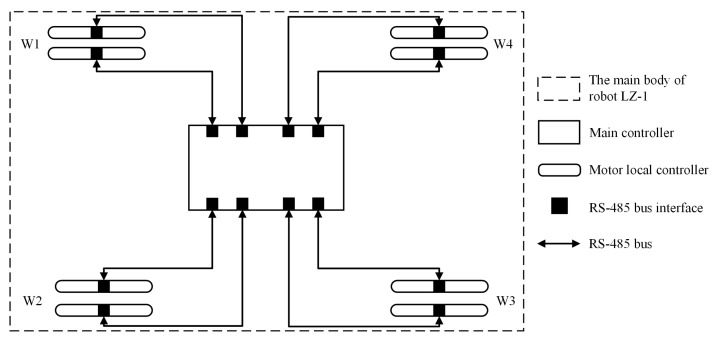
Controller structure diagram of LZ-1.

**Figure 11:**
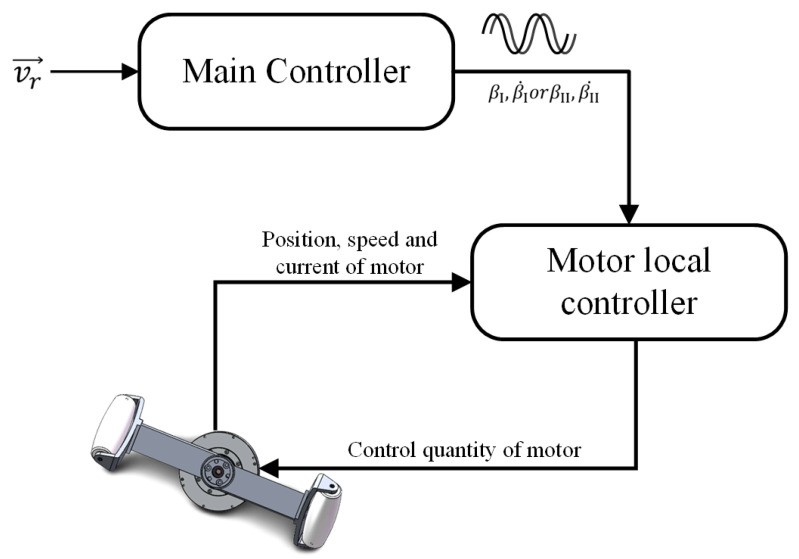
Control signal flow diagram from target speed of robot to single motor.

Figure 10 shows the relationship between the internal controllers of the LZ-1. The main controller is connected to eight local motor controllers through eight RS-485 buses. The communication bandwidth of the RS-485 bus was set to 2 Mbps. The main controller is mainly responsible for receiving the robot target velocity from the remote controller, calculating the position–time sequences or velocity–time sequences of the four FSM wheels’ spoke components in real-time according to the selected motion mode, and synchronizing these sequences to the corresponding servo motor local controllers. The servo motor local controller controls the motor to track the position or speed at the corresponding time in the position–time or velocity–time sequence. The main controller and the LZ-1 adopt an open-loop control method, while the motor local controller and motor adopt a closed-loop proportional–integral–differential (PID) control method. The local motor controller uses three-layer PID controllers to control the motor current, speed, and position, respectively, as shown in Figure 11.

## 3. Experiments and Results

In the experiment, we tested the LZ-1’s ability to move in all directions and adapt to different terrains in normal motion mode and its ability to traverse stairs in crawl motion mode.

**Figure 12:**
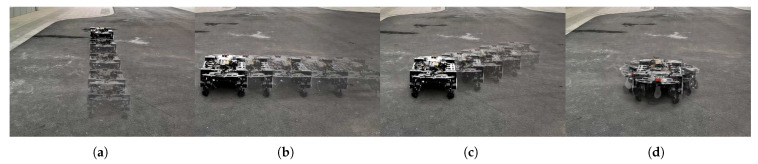
The trajectory of LZ-1’s omnidirectional motion mode. (**a**) The forward trajectory. (**b**) The lateral trajectory. (**c**) The oblique $-60^{\circ }$ motion trajectory. (**d**) The trajectory when rotating $360^{\circ }$.

In the experiment examining conventional motion mode, we tested the omnidirectional movement ability of the LZ-1 by testing its forward movement, lateral movement, $-60^{\circ }$ oblique movement, and $360^{\circ }$ rotary movement on flat ground. We used slow shutter photography to record the test process, and the test results are shown in Figure 12. Figure 12a shows the trajectory of the LZ-1 moving along the positive direction of the *x* axis of the robot body’s reference system (that is, in front of the robot) at a speed of 0.28 m/s. Figure 12b shows the trajectory of the LZ-1 moving along the positive direction of the *y* axis of the robot body’s reference system (that is, only to the left of the robot) at a speed of 0.18 m/s. Figure 12c shows the LZ-1 moving at a speed of 0.16 m/s along an angle of $-60^{\circ }$ on the *x* axis of the robot body’s reference system (that is, to the right and front of the robot). Figure 12d shows the LZ-1 rotating $360^{\circ }$ clockwise around its *z* axis. The LZ-1 can move at different speeds without adjusting its heading angle, proving its omnidirectional movement ability.

**Figure 13:**
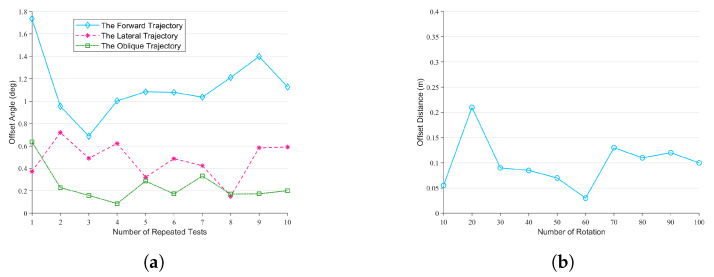
The error between the actual motion trajectories of LZ-1 and its target trajectories. (**a**) The angle of deviation between the forward, lateral, and diagonal motion trajectories of LZ-1 and its target trajectories. (**b**) The offset distance between LZ-1’s current position and its initial position after rotating by $360^{\circ }$.

In order to test the errors between the LZ-1’s actual trajectories and its target trajectories in the above four movements, we carried out many experiments. We carried out ten groups of experiments on the forward motion of the LZ-1’s omnidirectional motion mode. In each group of experiments, we let the LZ-1 move 5 m along the positive direction of its *x* axis at 0.28 m/s. Then, we tested the angle between its trajectory and its *x* axis at the initial time. We also carried out ten groups of experiments on the lateral motion of the LZ-1’s omnidirectional motion mode. In each group of experiments, we let the LZ-1 move 5 m along the positive direction of its *y* axis at 0.18 m/s. Then, we tested the angle between its trajectory and its *y* axis at the initial time. Similarly, we carried out ten groups of experiments on the oblique motion of the LZ-1’s omnidirectional motion mode. In each group of experiments, we let the LZ-1 move 5 m at 0.16 m/s along a $-60^{\circ }$-angle path on its *x* axis. Then, we tested the angle between its trajectory and the target direction. Figure 13a shows the results of these thirty groups of experiments, and the trajectories of the LZ-1 had errors in all directions. The performance of the forward movement was worse than that of the other two movements, but its maximum error was less than $2^{\circ }$.

In addition, we let the LZ-1 rotate around its *z* axis 100 times continuously and tested the offset distance between the current position and the robot’s initial position every 10 times. The experimental results are shown in Figure 13b. The experimental results showed that the rotation motion of the LZ-1 also had deviations, and its maximum deviation distance was 0.21 m.

Errors in the manufacturing and assembly of mechanical structures and errors in motor movement may cause these deviations. Therefore, we will study this problem carefully in the future and optimize the robot design to reduce deviations.

**Figure 14:**
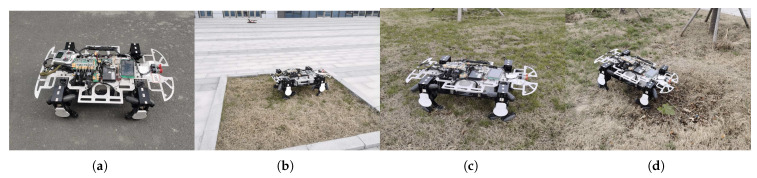
LZ-1 and four different road surfaces. (**a**) Paved road. (**b**) Flower bed. (**c**) Rough meadow. (**d**) Road with potholes.

We selected four typical outdoor surfaces to test the adaptability of the LZ-1’s omnidirectional motion mode on different terrains, as shown in Figure 14. Figure 14a shows a hardened pavement made of asphalt, which is very flat. Figure 14b shows a flower bed surrounded by gray bricks and a 12 cm-high border, and the surface is a soft soil layer. Figure 14c shows an undulating meadow with a slope of $40^{\circ }$. Figure 14d shows a deep 20 cm pothole with a radius of 40 cm. The LZ-1 can move through all four road surfaces in omnidirectional motion mode.

**Figure 15:**
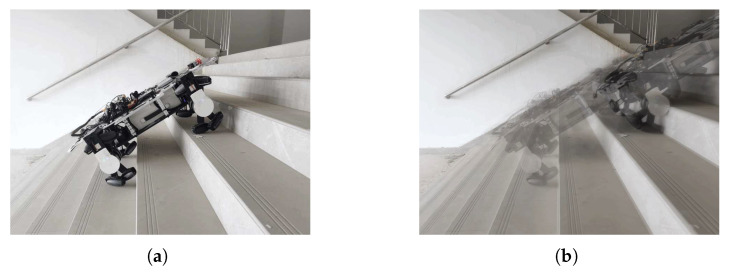
LZ-1 uses crawl motion mode to climb stairs.

In the experiment studying climbing motion mode, we chose a flight of stairs with ten steps to test the LZ-1. Figure 15a shows the stairs we used for testing, where a single step is 15 cm high and 28 cm wide, and the entire staircase is inclined at $30^{\circ }$. In the experiment, we tested LZ-1 on this staircase twenty times using its climbing motion mode, and it successfully climbed the stairs seventeen times. There were two times when the robot slipped down due to the rollers slipping and one time when the robot collided with the stair railing during climbing due to the robot’s initial heading offset. Figure 15b shows the trajectory of the LZ-1 during its climb on the stairs. Moreover, the entire video of the experiment can be found at the Supplementary Material.

## 4. Discussion and Conclusions

To solve the problem of the complex structure and lack of agility in hybrid mobile robots, we designed a simple structure called a flexible spoked mecanum wheel. Moreover, based on this structure, we designed a mobile robot named the LZ-1. We conducted detailed modeling analysis and control implementation for two typical motion modes of the LZ-1: the omnidirectional and the crawl motion modes. Through the experiments, we verified the omnidirectional movement ability of the LZ-1 with a simple movement structure, as well as its excellent locomotion on various complex terrains. The experimental results showed that our designed FSM wheel can build a mobile robot with excellent locomotion and agility through a simple combination, proving the effectiveness of the FSM wheel design and providing a new idea for the design of mobile robots. In summary, the LZ-1 mobile robot based on the FSM wheel had the following advantages:

1. The LZ-1 robot had omnidirectional mobility similar to traditional mecanum-wheeled mobile robots and a better obstacle-crossing ability. It can move on uneven terrain and climb continuous stairs.
2. Compared to the first type of hybrid mobile robot, the LZ-1 has a simpler structural design and uses fewer motors to move on various terrains. A typical wheel-legged hybrid robot requires about four motors for one leg, while our FSM-wheeled robot only needs two per leg.
3. Compared to the second type of hybrid mobile robot, the LZ-1 has a more straightforward process of changing motion modes. It can change between omnidirectional and crawl motion modes without changing the motion mechanism’s configuration.
4. The LZ-1 robot is a solution for mobile robots that balances design cost, control complexity, mobility, flexibility, and multi-terrain adaptability.

Of course, our design still needs some improvements. For example, the LZ-1 cannot turn in crawl motion mode, and it may still slip when climbing stairs. In future research, we will work hard to solve these problems and improve the design of the FSM wheel and the LZ-1. In addition, in the future, we will conduct more in-depth research on the LZ-1 and the FSM wheel with the following goals:

1. Improve the structural design and reduce possible errors;
2. Design more useful motion modes;
3. Study methods for switching between multiple motion modes;
4. Explore the impact of different loads on the robot’s movement capabilities;
5. Enable the LZ-1 to recognize terrain changes and switch motion modes autonomously.

## Figures and Tables

**Table 1:** The basic parameters of the FSM wheel.

Parameter Names	Symbols	Values	Unit
Half the length of the spoke component	*r*	0.160	m
Length of the roller	*l*	0.101	m
Diameter of the roller	*d*	0.036	m
Angle between the roller and the FSM wheel axes	*α*	−60 or 60	deg
Minimum included angle between the two spoke components	*β_min_*	20	deg

**Table 2:** The basic parameters of the LZ-1 robot.

Parameter Names	Symbols	Values	Unit
The length of LZ-1	*L*	0.950	m
The width of LZ-1	*K*	0.640	m
The height of LZ-1	*H*	0.335	m
The weight of LZ-1	*M*	47	kg
The front and rear FSM wheels’ distance	*F*	0.630	m
The left and right FSM wheels’ distance	*B*	0.489	m

## Data Availability

Not applicable.

## Supplementary Materials

The following supporting information can be downloaded at: https://www.mdpi.com/article/10.3390/biomimetics8020183/s1.
